# Randomized Controlled Trials to Treat Obesity in Military Populations: A Systematic Review and Meta-Analysis

**DOI:** 10.1192/j.eurpsy.2024.639

**Published:** 2024-08-27

**Authors:** D. Gravina, J. L. Keeler, M. N. Akkese, S. Bektas, P. Fina, C. Tweed, G. D. Willmund, L. Dell’Osso, J. Treasure, H. Himmerich

**Affiliations:** ^1^Institute of Psychiatry, Psychology and Neuroscience, King’s College London, Centre for Research in Eating and Weight Disorders, London, United Kingdom; ^2^University of Pisa, Department of Clinical and Experimental Medicine, Pisa, Italy; ^3^Bethlem Royal Hospital, South London and Maudsley NHS Foundation Trust, London, United Kingdom; ^4^Hacettepe University, Department of Psychology, Ankara, Türkiye; ^5^Sigmund Freud University Vienna, Faculty of Psychology, Vienna, Austria; ^6^Royal Navy Reserve, London, United Kingdom; ^7^Military Hospital Berlin, Bundeswehr Center for Military Mental Health, Berlin, Germany

## Abstract

**Introduction:**

In recent years, overweight and obesity have reached an alarmingly high incidence and prevalence worldwide; they have also been steadily increasing in military populations. Military personnel as an occupational group are often exposed to stressful and harmful environments that represent a risk factor for disordered eating with major repercussions on both physical and mental health.

**Objectives:**

This study aims to explore the effectiveness of weight loss interventions and to assess the significance of current obesity treatments for military populations.

**Methods:**

Three online databases (PubMed, PsycInfo, and Web of Science) were screened to identify randomized controlled trials (RCTs) aiming to treat obesity in active-duty military personnel and veterans. Random-effects meta-analyses were conducted for body weight (BW) and body mass index (BMI) values, both longitudinally comparing treatment group from pre-to-post intervention, and cross-sectionally comparing the treatment group to controls at the end of the intervention.

**Results:**

A total of 21 studies were included: 16 cross-sectional (BW: n=15; BMI: n=12) and 16 longitudinal (BW: n=15; BMI: n=12) were meta-analyzed, and 5 studies were narratively synthesized. A significant small overall BW and BMI reduction from baseline to post-intervention was observed (BW: *g* = -0.10; *p* = 0.015; BMI: *g* = -0.32; *p* < 0.001), together with a decreased BMI ( *g* = -0.16; *p* = 0.001) and nominally lower BW ( *g* = -0.08; *p* = 0.178) in the intervention group compared to controls at post-intervention time-point. When conducting additional meta-analyses dividing by sample type, a significant decrease in both BMI (g= -0.35; p< 0.001) and BW (g= -0.12; p= 0.041) from pre-to-post intervention was observed in active-duty military personnel but not for veterans.Recommendations for clinical practice have been outlined from the findings of this study and summarized in Figure 1.

**Image:**

**Figure:**
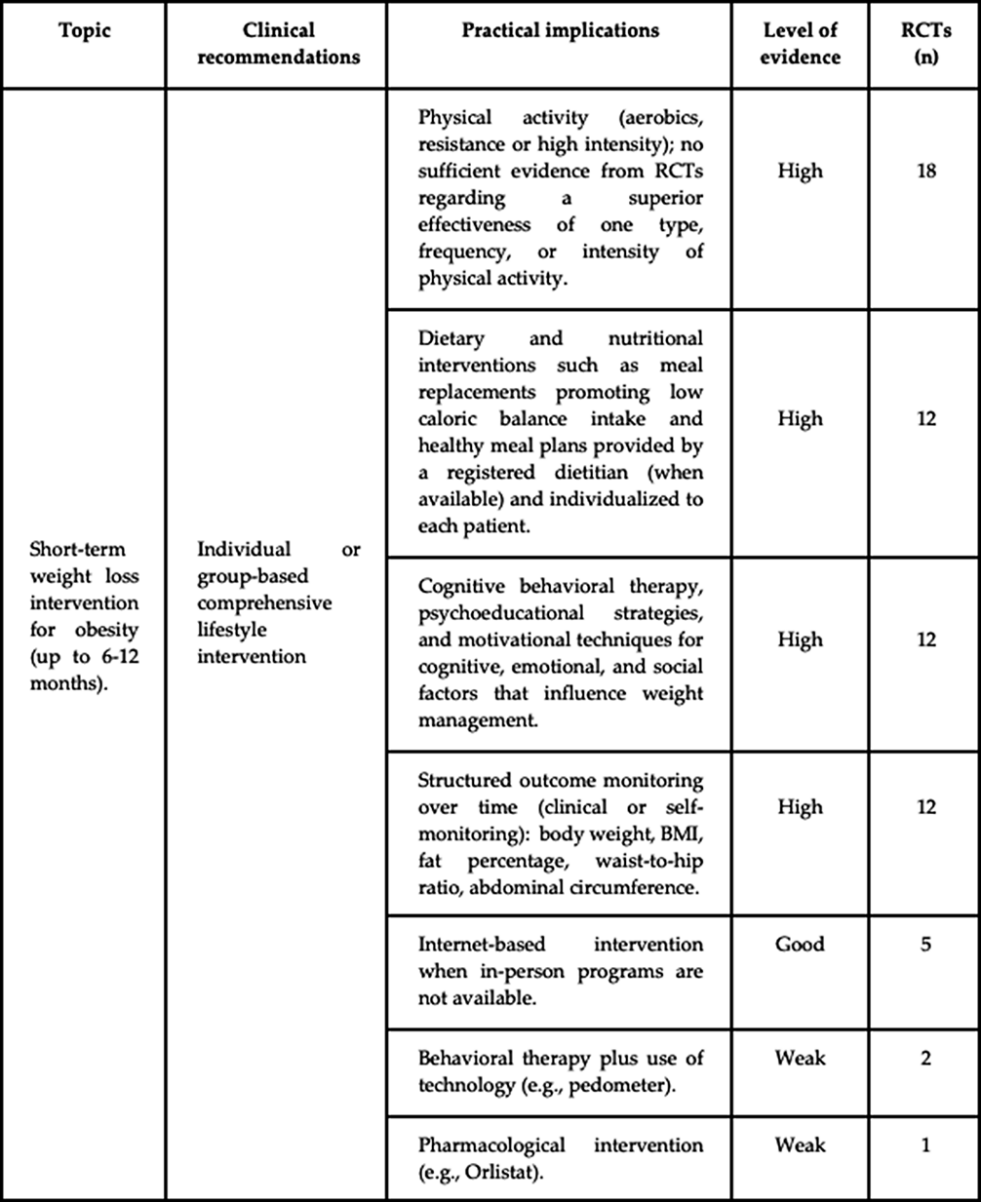

**Conclusions:**

Despite limitations, such as the heterogeneity across the included interventions and the follow-up duration, our findings highlight how current weight loss interventions are effective in term of BW and BMI reductions in military populations, and how a comprehensive approach with multiple therapeutic goals should be taken during the intervention.

**Disclosure of Interest:**

None Declared

